# Supplementary material for: Empirical methods for controlling false positives and estimating confidence in ChIP-Seq peaks
Source: BMC Bioinformatics. 2008 Dec 5;9:523. doi: 10.1186/1471-2105-9-523 (PMC2628906; doi:10.1186/1471-2105-9-523)
Supplement: Additional File 1 — A variety of html documents from the USeq web site detailing the available applications, their best usage, output file type descriptions, command line menus, etc. [file 1471-2105-9-523-S1.zip › USeqUserGuides/JavaDoc.zip!/JavaDoc/util/bio/annotation/class-use/TransGroup.html]

Uses of Class util.bio.annotation.TransGroup


|  |  |  |
| --- | --- | --- |
| **Overview** \| **Package** \| **Class** \| **Use** \| **Tree** \| **Deprecated** \| **Index** \| **Help** | |  |
| PREV   NEXT | **FRAMES**    **NO FRAMES**     **All Classes** |  |


---


## **Uses of Class util.bio.annotation.TransGroup**

| Packages that use TransGroup | |
| --- | --- |
| **gata.geneGlyphs** | For representing annotation, scale bars, and generic tracks. |
| **util.bio.annotation** | For representing gene annotation. |

Uses of TransGroup in gata.geneGlyphs

Constructors in gata.geneGlyphs with parameters of type TransGroup

`TransGrpGlyph(TransGroup transGrp,
GATAParams gataParams,
AnnoSpecParams annoParams)`

Uses of TransGroup in util.bio.annotation

| Methods in util.bio.annotation that return TransGroup | |
| --- | --- |
| `TransGroup[]` | `GeneGroup.getTransGrps()` |

| Methods in util.bio.annotation with parameters of type TransGroup | |
| --- | --- |
| `void` | `Translation.setTransGrp(TransGroup ref)`             Sets TransGroup ref but also fires the makeSegments method |

Constructors in util.bio.annotation with parameters of type TransGroup

`GeneGroup(java.lang.String name,
java.lang.String chromosome,
int start,
int end,
java.lang.String type,
TransGroup[] transGrps,
int orientation,
java.lang.String attributes)`

`GeneRep(TransGroup[] transGrps,
GeneGroup geneGrpRef)`

---


|  |  |  |
| --- | --- | --- |
| **Overview** \| **Package** \| **Class** \| **Use** \| **Tree** \| **Deprecated** \| **Index** \| **Help** | |  |
| PREV   NEXT | **FRAMES**    **NO FRAMES**     **All Classes** |  |


---
